# Report of Cases

**Published:** 1849-08

**Authors:** B. I. Raphael

**Affiliations:** One of the Attending Surgeons to the Louisville Marine Hospital


Art. IV.—*Report of Cases.* By B. I. Raphael, M. D., one of the Attending Surgeons to the Louisville Marine Hospital.

The singular autopsic appearances of the following cases have induced me to submit them to the profession.

*Case* 1. *Unique Malformation of an Infant—Autopsy.*—The subject under consideration, was a full sized, healthy looking infant, which at birth presented the following appearances: At the umbilical region there was a tumor about the size of a hen's egg, containing intestine. This could be easily reduced, but would return again as soon as the pressure was removed. There was a slight attempt at the formation of a penis and scrotum, but the testes had not descended; the anus was imperforate, and the posterior fontanelle wanting. The child lived nine days.

*Post Mortem Examination.*—The tumor situated at the umbilical region, contained nearly the whole mass of intestines, which were adherent to a considerable extent.

The entire colon was wanting. The extremity of the small intestines terminated in this umbilical sac, and meconium passed through an opening which had been made by ulceration. The left kidney was situated in its proper place, but the right one was found in the right hypogastric region. Both ureters terminated in the sac containing the intestines, and no trace of a bladder could be found. The sacrum, which was very broad, filled up the space between the rami of the ischium, and as the pubis was wanting, the coccyx, with the ascending rami of the ischium, formed the anterior part of the pelvis. The other organs appeared to be in their proper position.

*Case 2. Incised Wound of the Abdomen—Death—Autopsy.*—Joseph Smith, negro, aet. 26, waiter, about an hour before I saw him, received two incised wounds in the abdomen, one on each side of the median line. The one on the right side was half way between the umbilicus and pubis, and one inch external to the median line. The one on the left was on a line with the umbilicus, just over the point of the eleventh rib. Both of these wounds were transverse, and about half an inch in length, and had penetrated into the cavity of the abdomen. A small portion of omentum protruded through the wound on the left side. There was also a contusion of the nose and the left eye, and the patient was in a state of intoxication; he complained of great pain in his bladder, and was soon after seized with vomiting. A catheter was introduced into his bladder, but no water came away. The wounds were dressed—the one on the left side with a suture and adhesive strap; the one on the right side with straps alone. Soon after this he fell asleep.

April 10th. Patient evinces symptoms of peritonitis; his pulse was small and tense, and he complained of great pain over the whole abdominal region, but more especially near the wound on the right side; a whitish fluid, tinged with blood, flowed freely through this wound when slight pressure was made on the abdomen. Three other wounds were also discovered: one situated near the coracoid process of the left scapula, the others on the left side of his back—the superior one corresponding to the external edge of the scapula near its middle; the inferior one between the tenth and eleventh ribs four inches from the spine. The edges of these wounds were drawn together by adhesive straps. Vs. ℥ xij. and a simple injection given. Twelve leeches were also applied to the abdomen, and the bleeding to be kept up, with hot fomentations. These means, however, proved unavailing; the patient continued to sink during the day, and died at 9½ o'clock, P. M.

*Autopsy, twelve hours after death.*—When the abdomen was opened, the wound on the anterior part of the body, over the eleventh rib, was found to be larger internally than externally—that on the tegumentary surface being about half an inch in length; that on the peritoneal surface one and a half inches. The one on the right side was about in the same state, and had reached the jejunum about its middle. The wound of the jejunum was a transverse one, about three-fourths of an inch in length, with everted and gaping edges. A considerable quantity of feculant matter had made its way into the cavity of the abdomen through this opening. The cavity of the thorax on the left side, contained about ℥vi. of effused blood. The organs, generally, were healthy. The wound over the coracoid process of the scapula passed obliquely downwards, backwards, and outwards, almost to the shoulder joint, just escaping the axillary artery and brachial plexus of nerves. The superior wound on the back passed about an inch and a half downwards, forwards, and inwards, into the substance of the infra spinatus muscle. The wound between the tenth and eleventh ribs passed downwards through the lung about one inch and a half from its base, through the diaphragm and into the substance of the spleen, a small portion of the tenth rib having been broken off by the blow.

*Case 3. Incised Wound of the Diaphragm and Stomach.*—I was called, November 7th, 1848, about 7 o'clock, to see Henry Bremaker, a lad, aged 19, who was wounded in a fight, about ten minutes previous; found him lying in the barroom of a coffee house, and on examination discovered a wound about one inch in extent, through which a small portion of omentum protruded, situated on the left side, between the eighth and ninth ribs, and about midway between the sternal and vertebral articulations.—The finger could easily be passed through the wound into the cavity of the abdomen. A quantity of beans and other ingesta were found on the surface of the abdomen, which, as no vomiting had occured, left no doubt that the stomach was also wounded. The patient suffered much pain; his pulse was considerably accelerated, but not wanting in force.

*Treatment.*—After returning the protruded portion of the omentum, the edges of the wound were brought together and retained by strips of adhesive plaster, together with a compress and roller. The patient was then placed on a litter, conveyed home, and thirty drops of laudanum administered, with directions to repeat the dose in three hours, if necessary.

10, P. M. Was called to dress a small wound on the left arm, just above the elbow, which had been overlooked in the previous examination. On enquiry, learned, that the patient had vomited several times, and had thrown up a small quantity of blood. Pulse about the same as in the early part of the evening. As he complained of excessive thirst, and had drank much water, ice was ordered to be given in small quantities.

Nov. 8th. Saw patient at 6 A. M. Found him breathing with much difficulty, in consequence of which the bandage and compresses were removed. About half a pint of thin dark colored fluid gushed from the wound; he expressed himself much relieved, but his pulse gave evident indication of approaching dissolution.

9 A. M. More feeble; pulse scarcely perceptible. He continued to sink and died at 11½, A. M.

*Autopsy*, 3½ *hours after death.*—The external wound was one inch in length, transversely; midway between the spine and sternum; six inches below the axilla, and between the eighth and ninth ribs, making a wound in the intercostal muscles of two and a quarter inches, and extending to within an inch of the corresponding cartilages; passing through the cordiform tendon of the diaphragm, leaving an opening sufficiently large for the passage of the entire stomach, and a part of the transverse colon, together with the omentum. The stomach was perforated at the great cul-de-sac—the opening being about three fourths of an inch long, and situated about four inches from the æsophageal extremity, the mucous membrane everted and of a deep cherry color. The stomach contained half a pint of ingesta. The left lung completely collapsed and of a bluish macerated appearance. The left pleuritic cavity contained about a pint of bloody fluid, composed in a great measure of the contents of the stomach. The heart and right lung normal, as were also all the abdominal and pelvic viscera, with the exception of the stomach. A small quantity of bloody serum found in the convolutions of the intestines and pelvis. No peritoneal inflammation.

